# PBAT/PLA-Based Electrospun Nanofibrous Protective Clothes with Superhydrophobicity, Permeability, and Thermal Insulation Characteristics for Individuals with Disabilities

**DOI:** 10.3390/polym16172469

**Published:** 2024-08-30

**Authors:** Muhammad Omer Aijaz, Ubair Abdus Samad, Ibrahim A. Alnaser, Md Irfanul Haque Siddiqui, Abdulaziz K. Assaifan, Mohammad Rezaul Karim

**Affiliations:** 1 Center of Excellence for Research in Engineering Materials (CEREM), Deanship of Scientific Research (DSR), King Saud University, Riyadh 11421, Saudi Arabia; maijaz@ksu.edu.sa (M.O.A.); uabdussamad@ksu.edu.sa (U.A.S.); ianaser@ksu.edu.sa (I.A.A.); 2 King Salman Center for Disability Research, Riyadh 11614, Saudi Arabia; msiddiqui2.c@ksu.edu.sa (M.I.H.S.); aassaifan@ksu.edu.sa (A.K.A.); 3 Department of Mechanical Engineering, College of Engineering, King Saud University, Riyadh 11451, Saudi Arabia; 4 King Abdullah Institute for Nanotechnology, King Saud University, Riyadh 11451, Saudi Arabia; 5 Biomedical Technology Department, College of Applied Medical Sciences, King Saud University, Riyadh 12372, Saudi Arabia

**Keywords:** protective clothes, PBAT/PLA, disability, nanoparticles, nanofibrous membrane, hydrophobicity, thermal insulation, breathability

## Abstract

This study presents the development of multifunctional protective clothing for disabled individuals using PBAT/PLA biopolymeric-based electrospun nanofibrous membranes. The fabric consists of a superhydrophobic electrospun nanofibrous cloth reinforced with silica nanoparticles. The resulting nanofiber membranes were characterized using FE-SEM, a CA goniometer, breathability and hydrostatic pressure resistance tests, UV–vis spectroscopy, thermal infrared photography, tensile tests, and nanoindentation. The results demonstrated the integration of superhydrophobicity, breathability, and mechanical improvements in the protective clothing. The nanofibrous porous structure of the fabric allowed breathability, while the silica nanoparticles acted as an effective infrared reflector to keep the wearer cool on hot days. The fabric’s multifunctional properties make it suitable for various products, such as outdoor clothing and accessories for individuals with disabilities. This study highlights the importance of selecting appropriate textiles for protective clothing and the challenges faced by disabled individuals in terms of mobility, eating, and dressing. The innovative and purposeful design of this multifunctional protective clothing aimed to enrich the lives of individuals with disabilities.

## 1. Introduction

Poly(butylene adipate-co-terephthalate) (PBAT) and polylactide (PLA) are both polymers that may undergo biodegradation [1,2,3]. These are thermoplastics that can be manipulated using standard polymer processing techniques. PLA has high tensile strength and modulus, measuring 63 MPa and 3.4 GPa, respectively. However, it is brittle, with a strain at breaking of 3.8% [3]. On the other hand, PBAT is characterized by its flexibility and toughness, with a strain at breaking of 710% [4,5]. Considering their mutually beneficial characteristics, combining PLA with PBAT is an obvious decision to enhance the features of PLA while maintaining its biodegradability. PBAT is regarded as the most practical replacement for low-density polyethylene (LDPE), even though it is synthesized from petrochemical resources [6,7,8]. Even though PBAT first hit the market about 10 years ago, its poor strength and high manufacturing costs have made it less popular than LDPE, the most frequently used packaging film material [9]. Adding inexpensive additives to the polymer matrix, including PLA, starch, CaCO_3_, and hydrotalcite, often improves the mechanical characteristics of PBAT films [10,11,12,13].

Clothing has a multitude of purposes for humans, including protection against weather, security, comfort, and self-representation [14]. Clothing that meets an individual’s requirements might increase confidence in their social interactions. Unfortunately, more than one billion disabled persons globally do not receive enough medical or psychological care [15]. People with impairments and the elderly may enhance their quality of life by choosing more visually beautiful and useful textile goods. However, many circumstances have restricted this option. People with disabilities have a broad variety of unique needs compared with the general population, and there is typically a restricted local market for specialized things.

Furthermore, impairments typically create particular practical requirements for textiles and clothes. Individuals with highly sensitive skin should avoid clothes with rough seams. People who spend lengthy amounts of time in bed, as well as wheelchair users, need a temperature balance between the environment and the body. In general, it is difficult to maintain an ideal thermal balance, particularly for those with low heat production due to inactivity; hence, thermal comfort elements are important [16]. The transfer of moisture (sweat) from the skin to the body is a critical issue. Transfer of water vapor is important in assessing the breathability of clothing for both indoor and outdoor usage. Excess heat is eliminated from breathable garments by evaporating the moisture via micropores. If the fabric is made of nonbreathable materials, such as plastic, thick multilayered textiles, or fabrics with a thick pile, the body will prevent heat and moisture from being absorbed into the skin and the garments. Heat accumulates inside the body when layers of clothing are impermeable and trap moisture between the skin and the garment. As a consequence, heat and moisture accumulate, causing pain, wet skin, and skin abrasion. This may stimulate the activity of Gram-negative bacterial germs, which increases the chance of developing decubitus [17]. Some persons need clothing materials with exceptional tactile properties due to their extraordinarily sensitive skin. Breathability and waterproofness are important properties of both indoor and outdoor apparel. Fabrics constructed of nanofibrous (NF) materials may remove liquid moisture from the skin. The porosity of fibers may be altered by adjusting their diameter [18,19].

Individuals, particularly children, the elderly, and people with disabilities, may struggle to conduct self-care chores, such as eating, dressing, and moving about, owing to tremors or poor motor coordination. Stains from food and beverage spills may be difficult to remove from garments and cause skin discomfort. As a result, it is essential to carefully choose fabrics for protective clothes, notably bibs or aprons used when feeding. In hot weather, protective apparel must have high thermal insulation properties, which vary depending on the amount of activity and outside temperature. Two forms of ultraviolet radiation (UV) have been linked to an increased risk of skin cancer. There are two forms of UV radiation: UVA and UVB. UVA has a long wavelength of 315–400 nm and is connected with skin aging; UVB has a shorter wavelength of 280–315 nm and is associated with skin burns. In hot areas where the temperature increases fast and ultraviolet (UV) radiation may cause skin burns, insulation refers to the capacity to keep the body cool.

To provide the best protective efficacy, textiles must have a porous structure that allows air and water vapor to flow through while rejecting liquid water. The ideal protective materials for individuals with disabilities should be lightweight, air-permeable, water-repellent, breathable, capable of absorbing liquid moisture, and with good thermal insulation capabilities.

The aforementioned properties of hydrophobicity, waterproofness, breathability, and UV resistance can be incorporated into protective clothing using a variety of techniques, including template methods, fiber fibrillation techniques, melt-blowing processes, phase separation methods, and electrospinning [20,21,22,23,24]. Electrospinning has been shown to be an excellent method for creating multifunctional protective garments. This approach creates a microporous NF structure composed of a linked porous network of fibers. The resulting electrospun sheets may be used as textile protectors, and the membrane structure can be changed by varying the fibers’ diameter [20,25,26,27]. The creation of one-layer microporous electrospun membranes that are both waterproof and breathable has resulted in significant breakthroughs [28,29,30,31]. Mohsen et al. used two opposite-nozzle electrospinning setups to create a dual-functional NF membrane with one side made of polyurethane and the other side made of poly(2-acryloylamido-2-methylpropanesulfonic acid)-graphene oxide (PAMPS-GO). Researchers investigated the water vapor permeability of PAMPS nanofibers and observed that increasing the concentration of graphene oxide improved the permeability. Yuliang et al. performed a similar study to investigate the behavior of liquid moisture transport across an electrospun membrane. They created a dual layer made of polyacrylonitrile (PAN) and polystyrene (PS), which was then modified with polydopamine coatings to produce a much drier inner layer. The scientific community focused on modern membrane technology is fully aware of the negative effects of using petroleum-based polymers on the environment and human health. Biodegradable polymers have lately gained popularity due to their biocompatibility and environmental advantages over petroleum-based polymers [22,32]. Polylactic acid (PLA) is a biodegradable and biocompatible biopolymer with a distinct chemical, thermal, and mechanical profile. It is a hydrophobic, thermoplastic biopolymer with high durability, flexibility, and resistance to UV light. As a result, PLA has emerged as a very sustainable and promising natural fiber material for use in protective gear. PLA-based electrospun nanofibers are gaining popularity for medical implants, wound dressings, water purification, scaffolds, delivery systems for medication, fog collecting, and surgical suture applications. This is owing to their capacity to closely replicate the extracellular matrix, provide a large specific surface area, display high porosity with a tiny pore size, and have acceptable mechanical characteristics [33,34,35,36,37,38]. Previous studies have focused on the use of PBAT to improve the brittle and hard nature of PLA. As both polymers are biodegradable and biocompatible, blending them has been extensively studied in the form of sheets and films [39,40,41]. However, limited applications were found for the material, as it is nonwoven [42,43,44].

This work is the first to use a sequential electrospinning setup to create PBAT/PLA electrospun NF textiles with a superhydrophobic waterproof surface. In our design, the superhydrophobic layer was made of pure PBAT and PLA solutions with SiO_2_ nanoparticles (NPs). The addition of PBAT to PLA materials eliminated the drawbacks of PLA and resulted in the production of materials with enhanced mechanical properties. Furthermore, the incorporation of hydrophobic SiO_2_ endowed the material with the ability to reflect ultraviolet radiation, thereby offering protection for the wearer’s skin against ultraviolet exposure and providing high water repellency. The morphological and wettability changes in the hydrophobic protective cloths were validated using field emission scanning electron microscopy (FE-SEM) and contact angle (CA) tests. Furthermore, tests were performed to assess the protective features in terms of breathability, hydrostatic pressure, and capability for protection against sunlight.

## 2. Experiments

### 2.1. Materials and Methods

The core elements of nanofibrous textiles were made from polybutylene co-adipate co-terephthalate (PBAT, trade name: BG1070; MFI 2–5 g/10 min, density 1.25 g/cm^3^), acquired from Ankor Bioplastics, LTC (Wonjo, Republic of Korea), and polylactic acid (PLA; LX175^®^, Filabot, Barre, VT, USA). Silica nanoparticles were embedded into textiles purchased from Sigma Aldrich. Sigma Aldrich (St. Louis, MO, USA) provided dichloromethane (DCM) and dimethylformamide (DMF) for preparation of the electrospinning solution. The hydrophobicity of the textiles was tested using deionized water.

### 2.2. Preparation of PBAT/PLA-Based Multifunctional Electrospun Fabrics

The following steps were performed to produce electrospun nanofiber (NF) fabrics with properties such as hydrophobicity, UV protection, breathability, and mechanical stability. First, separate solutions of PBAT and PLA were prepared in a binary solvent consisting of dichloromethane (DCM) and N,N-dimethylformamide (DMF) in a 4:1 ratio for 60 min at 50 °C. The PBAT solution had a concentration of 15 wt.%, whereas the PLA solution had a concentration of 12 wt.%. The PBAT and PLA solutions were homogeneously combined in a 3:1 ratio, respectively, using a magnetic stirrer for 90 min at 50 °C, resulting in a solution for the nanofibrous membrane. The solution was then mixed overnight at room temperature with various concentrations of silica. Sonication was performed for 30 min to improve the nanoparticles’ dispersion and remove bubbles from the solution. The resulting solutions were labeled according to the SiO_2_ content, as indicated in Table 1 (referred to as M1, M2, M3, and M4 for silica concentrations of 1, 3, 5, and 7%, respectively).

### 2.3. Production of Electrospun Nanofiber Fabrics

Nanofibers were produced at CEREM, KSU using an electrospinning device (model NF-500, MECC, Fukuoka, Japan) capable of delivering 30 kV power. The PBAT/PLA solution containing various nanoparticles was fed into a 10 mL syringe with a needle 0.6 mm in diameter, which was then connected to a pump assembly and a high-voltage supply. The distance between the needle and collector was 15 cm, with an applied voltage of 18 kV and a flow rate of 0.8 mL/h. The humidity was maintained at 10%, and the resulting nanofiber membranes were dried for 24 h at 60 °C in an oven. Finally, the composite textiles were labeled according to the information provided in Table 1.

### 2.4. Characterizations

FE-SEM was utilized to examine the microstructure of the electrospun fabrics. The JEOL (JSM-7600, Tokyo, Japan) device was used for this investigation. Tiny specimens of cloth were cut and affixed to a carbon-coated stub using carbon tape. The stub was subsequently coated with platinum, which improved the electrical conductivity of the sample during analysis. Ultimately, FE-SEM analysis of the coated samples was conducted in a high-vacuum environment.

The thermal characteristics of the prepared fabrics were investigated using a thermal gravimetric analysis (TGA) machine (Q600, TA Inc., New Castle, DE, USA) in the temperature range of 25 to 600 °C at a heating rate of 20 °C/min. The analysis was conducted under an inert atmosphere (N_2_ gas) using a small amount of electrospun nanofibers placed in a ceramic pan. The crystallinity and structure of the protective cloth were examined via X-ray diffractometry (XRD) (XRD-7000, Shimadzu, Kyoto, Japan) in the 2–60 2θ-degree range, with continuous scanning at a rate of 2°/min.

The wettability behavior of the electrospun fabrics was evaluated using a CA goniometer, specifically, the OCA 15EC model from Data Physics. The angle at which a droplet of deionized water made contact with the NF textile was measured to determine its wettability. The analysis was conducted at multiple locations on each sample, and the results were averaged to obtain a representative value. The breathability and resistance to hydrostatic pressure of the PLA-based membranes were evaluated using a manual testing setup. To assess the movement of air through the nonwoven fabrics, a conical flask, an airflow meter, and a compressed air supply were used in a breathability test. The resistance to hydrostatic pressure of the cloth was evaluated in accordance with the ISO 811 standard [45] by using a manual tester with a diameter of 4 cm constructed in the laboratory. The temperature of the test water was maintained at 20 °C, and the relative humidity of the test room was set to 20%. The pressure on the cloth’s surface was gradually increased using a vacuum pump until a water drop appeared, with the rate of the increase in pressure set at 2 cm Hg.

The evaluation of the heat barrier qualities of the electrospun fabric was carried out using UV–vis spectroscopy to determine the percentage of reflection. A UV–vis–NIR scanning spectrophotometer (UV3600, Shimadzu, Kyoto, Japan) equipped with an integrating sphere attachment, suitable for measuring wavelengths ranging from 200 to 800 nm, was used to assess the optical properties of the electrospun NF fabrics. To monitor the thermal behavior of the materials, thermal infrared photographs of the electrospun protective clothing were visually observed when exposed to direct sunlight. The heating responses of the materials were recorded using an IR camera (FLIR, E64501, Wilsonville, OR, USA).

Tensile tests for the membranes were carried out using an Instron apparatus (Electropuls, UK). The gauge length of the samples was 20 mm × 45 mm at a testing rate of 5 mm/min. To facilitate handling, all samples were adjusted in a paper frame, and after the specimens had been gripped by the machine, the paper frame was cut with scissors, leaving only the NF fabrics between the grips. The average values of the ultimate tensile strength and elongation at breaking were analyzed from the stress–strain curve. The textiles’ surface hardness was measured using a nanoindentation machine. The indentation properties of the nanofibrous textiles were evaluated with the help of a Berkovich-type indenter using a Nanotest-3 platform (Micromaterials, Wrexham, UK). To determine the properties, indentation was executed using the depth control method, and the maximum penetration depth for each sample was set to 5000 nm. All tests were performed at a loading rate of 0.05 mNs^−1^ because of the samples’ soft nature, followed by a similar unloading rate until the load was completely removed. At least 10 indentations were performed on each sample to obtain uniformity in the results, although the final results presented have been averaged.

## 3. Results and Discussion

### 3.1. Preparation of Superhydrophobic Electrospun Nanofibrous Fabrics

**Figure 1:**
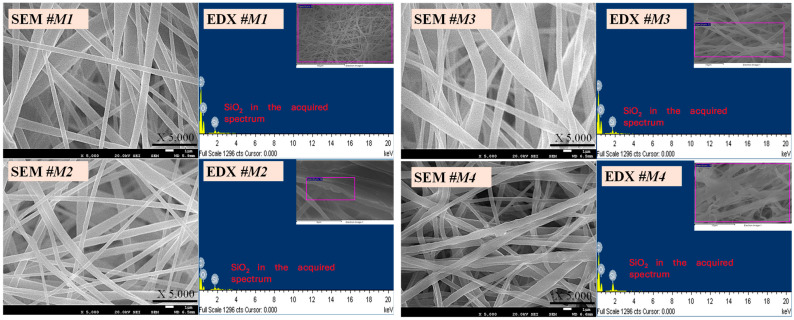
FE-SEM and EDX images of PBAT/PLA-based multifunctional electrospun fabrics.

Figure 1 exhibits the morphological images in the PBAT/PLA multifunctional hierarchically constructed nanofibrous textiles, accompanied by EDX data confirming the presence of silica nanoparticles. All four samples, namely M1, M2, M3, and M4, exhibited comparable fiber architectures and nanoparticle characterizations. The tiny PBAT/PLA nanofiber fibers shown in the study were characterized by their smoothness and lack of beads or drops. The calculated mean diameters for M1, M2, M3, and M4 were 860, 776, 810, and 615 nm, respectively, according to the built-in SEM software. The decrease in the mean diameter with the increase in the concentration of SiO_2_ was attributed to the repulsive force between the nanoparticles, which reduced the entanglement of the polymer chains [46,47].

### 3.2. Thermal Analysis

**Figure 2:**
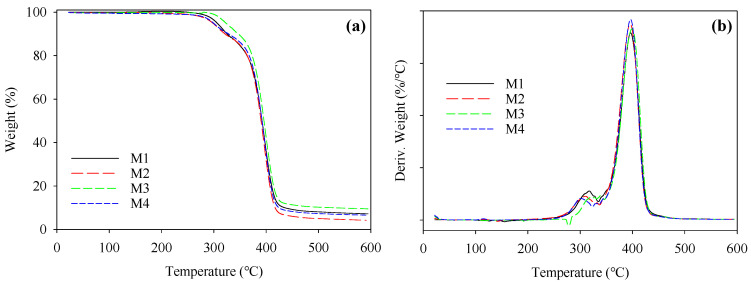
Thermal analysis of PBAT/PLA-based multifunctional electrospun fabrics to show the thermal stability of the (**a**) TGA and (**b**) DTA data.

Figure 2a,b show the TGA and DTA curves from the thermal analysis of the PBAT/PLA-based multifunctional electrospun textiles, respectively. The PBAT/PLA membranes’ initial decomposition temperature was 339.7 °C, and their maximum breakdown rate occurred at 357 °C. The PBAT/PLA fibers started to degrade at around 321 °C and achieved their maximal thermal breakdown rate at about 347 °C. It can be observed that the degree of crystallinity of PBAT/PLA materials was proportional to their thermal stability, with the thermal stability rising as the crystallinity grew. As a consequence, rapid crystallization occurred in the PBAT/PLA membranes during the electrospinning process; however, the PBAT/PLA molecular chains were not sufficiently altered during electrospinning, resulting in decreased crystallinity and thermal stability [19].

### 3.3. X-ray Diffractometry (XRD)

**Figure 3:**
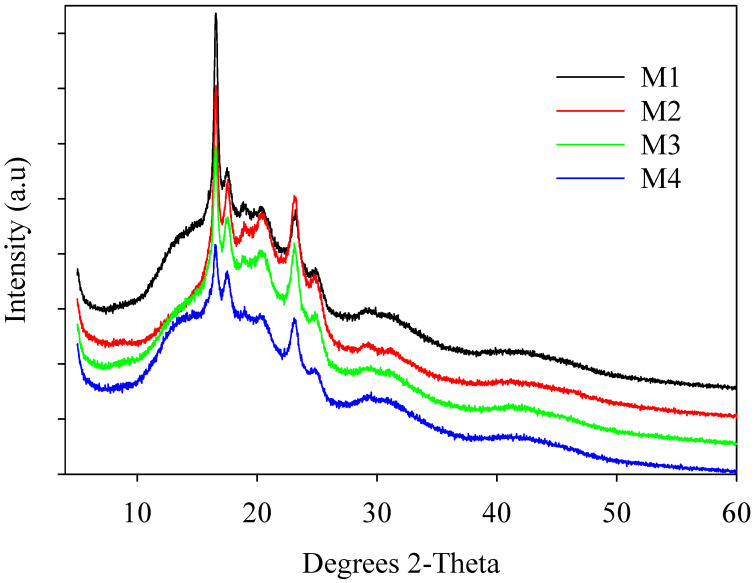
XRD patterns showing characteristic peaks of PBAT/PLA-based multifunctional electrospun fabrics.

We also used XRD to study the crystal properties of the PBAT/PLA blends (Figure 3). The XRD curves for the PBAT/PLA-based multifunctional electrospun textiles revealed prominent diffraction peaks at 2*θ* = 18.7°, corresponding to the (203) crystal plane [19,48]. The XRD peaks for the PBAT/PLA blends were almost identical to those for PBAT and PLA (not included here), but the diffractive intensities of the blends were reduced due to a low PLA content. Several tiny diffraction peaks were found in all samples, perhaps connected to the microcrystallinity of the PBAT, PLA, and silica NPs.

### 3.4. Wettability Study

**Figure 4:**
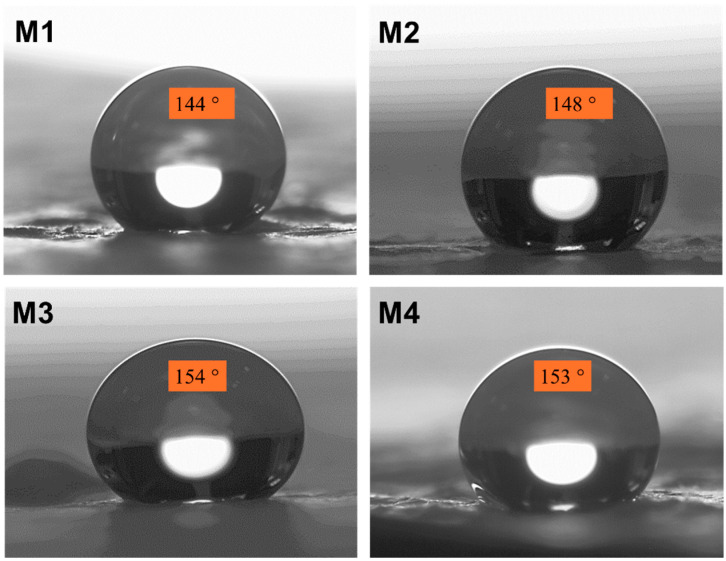
CA images of PBAT/PLA-based multifunctional electrospun fabrics to prove their resistance to wettability.

Figure 4 depicts the CA measurements taken for the generated NF fabric to establish the nature of the electrospun textiles. It shows the high and consistent contact angle of up to 154° for the M3 sample. The shape of the water drop stayed unchanged throughout the test, as seen in the image. This superhydrophobic property confirmed the waterproofness of the electrospun materials. The protective layer’s increased hydrophobicity protects a person’s skin and clothes from being discolored when they accidently spill drinks or when mud splashes from the road on rainy days.

### 3.5. Measurements of Breathability and Hydrostatic Pressure 

**Figure 5:**
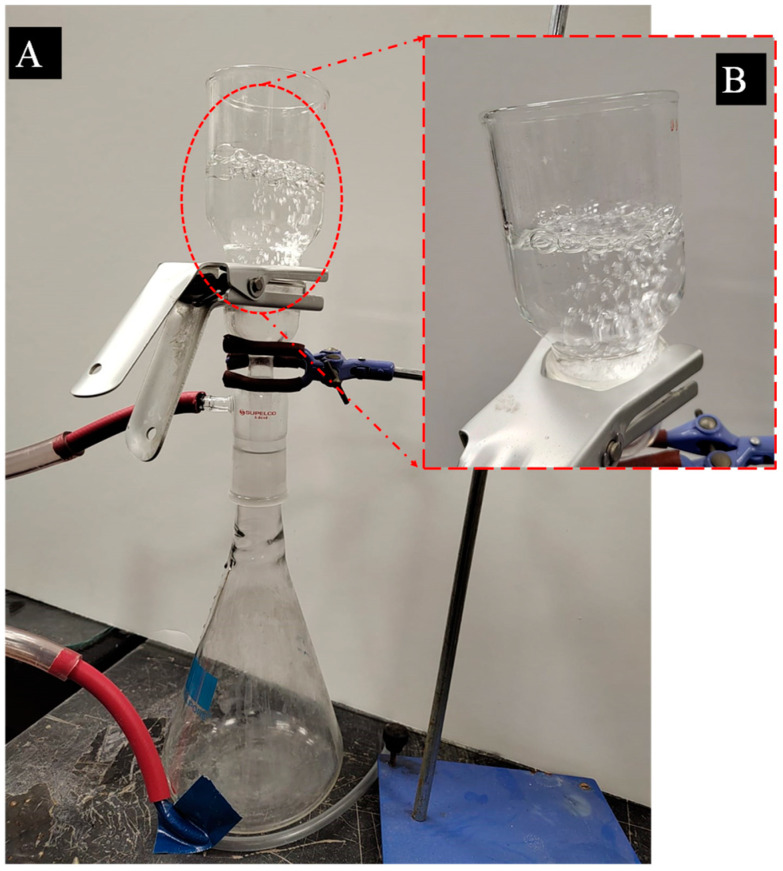
Images of the breathability test of the PBAT/PLA-based multifunctional electrospun fabrics: (**A**) the breathability test’s setup; (**B**) zoomed-in image of the cell to show the bubbles.

SiO_2_ NPs were present on the fabric layer of electrospun textiles. It was imperative to examine the influence of these layers on the permeability of the fabric, as NPs have the potential to occupy the spaces between the NFs’ structures. In order to verify their efficacy, the fabrics’ waterproofness and air permeability (breathability) were assessed and analyzed through a series of experiments, as illustrated in Figure 5. Bubbles of liquid began to emerge, as illustrated in the figure. The bubbles rapidly expanded as the air gas supply began, indicating that the permeability was satisfactory.

**Figure 6:**
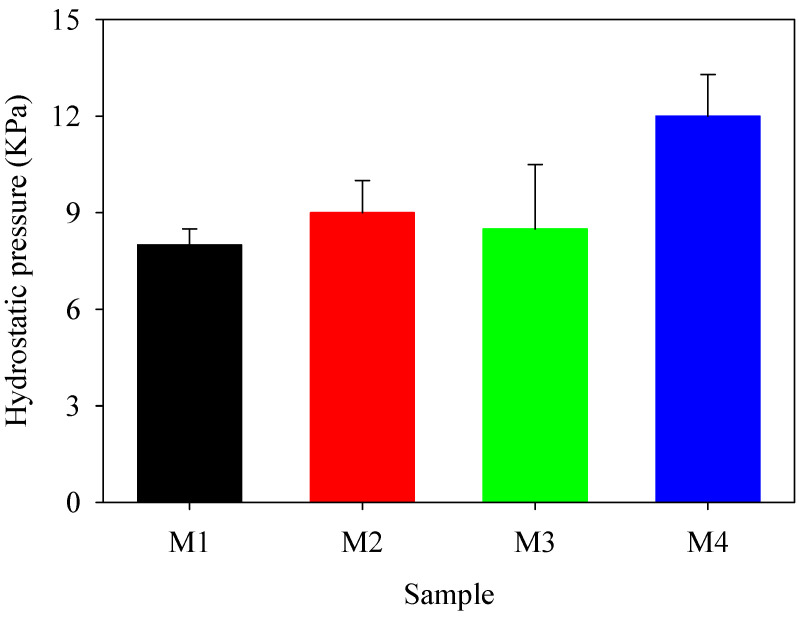
Hydrostatic test results showing water barrier properties of PBAT/PLA-based multifunctional electrospun fabrics.

The membranes underwent hydrostatic testing to further confirm their waterproofness and strength. Figure 6 illustrates the fabric’s qualities as a hydrostatic barrier. As the pressure increased, the water pressure applied to the membrane’s surface likewise increased. The water pressure exhibited a progressive rise, eventually reaching a threshold of 24 cm Hg, resulting in the appearance of discernible water droplets on the opposite side of the membrane.

### 3.6. Heat Reflector

**Figure 7:**
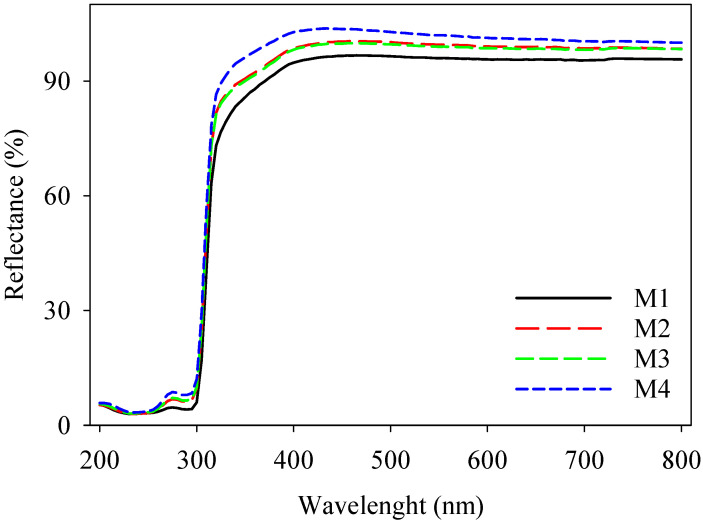
UV–vis curves of PBAT/PLA-based multifunctional electrospun fabrics, showing UV protection.

The UV–vis and IR imaging techniques were used to verify the UV reflection properties of the electrospun textiles that were manufactured. Figure 7 exhibits the reflection of the superhydrophobic fabric over a range of wavelengths from 200 nm to 800 nm. The SiO_2_ nanoparticles in the superhydrophobic layer effectively blocked UV radiation in both the UV-A (315–400 nm) and UV-B (280–315 nm) zones, with blocking percentages of 74% and 78% respectively. When comparing the textiles with pure PLA, the average reflectance in the visible wavelength range (400–800 nm) was 78% greater [45]. The resulting protective fabric exhibited superior performance in terms of reflection (%) due to its ability to reflect UV rays in the 280–400 range. This would not only help to keep the body temperature cool but would also aid in preventing skin burns, aging, and ultimately, skin cancer [18].

**Figure 8:**
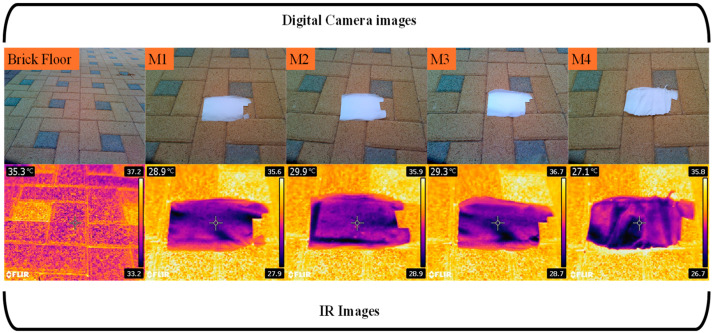
Digital and infrared images of a brick floor with and without PBAT/PLA-based multifunctional electrospun fabrics in bright sunlight.

Moreover, infrared camera photos were acquired to evaluate the thermal effects of the PBAT/PLA protective fabrics when exposed to direct sunshine. Figure 8 displays both real-time and infrared photos of a brick floor exposed to direct sunlight outside. The photographs compare the floor with and without protective clothing made of electrospun NFs. The temperature of the uncovered floor in the photographs was recorded at 35.5 °C. Nevertheless, as shown in the figure, the temperature of the bricked floor which was shielded by protective clothing exhibited a noteworthy decrease. The measurements recorded for the M1, M2, M3, and M4 samples were 28.9, 29.9, 29.3, and 27.1 °C, respectively. This protective garment led to a significant decrease of up to 24% in the floor’s temperature when exposed to direct sunlight.

### 3.7. Mechanical Strength

**Figure 9:**
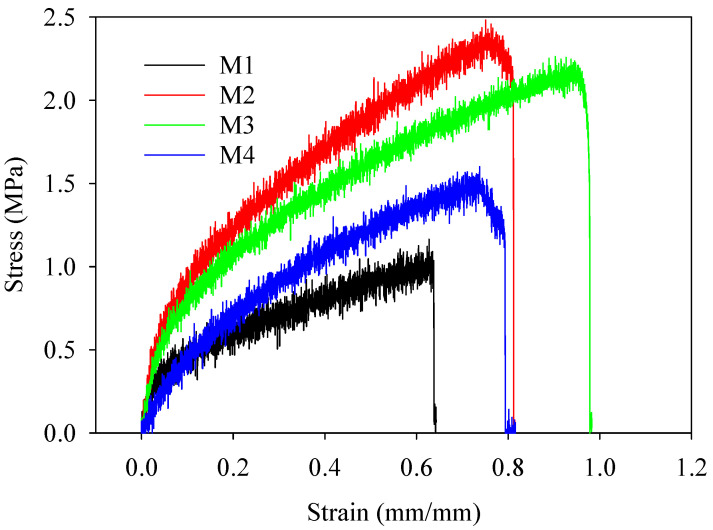
Tensile test curves showing the mechanical behavior of PBAT/PLA-based multifunctional electrospun fabrics.

An analysis was conducted on the tensile strength of the multifunctional electrospun textiles made from PBAT/PLA. The stress–strain curve shown in Figure 9 was used for this analysis. Tensile experiments provided an understanding of the overall properties of the electrospun nanofiber garments. First, the membranes displayed a straight-line pattern and had a somewhat steep incline. Then, in the subsequent session, the level of stress was increased in a consistent and progressive way. During the last session, there was a rapid occurrence of breaking, resulting in a significant decrease in stress. The fabric had a maximum ultimate tensile strength of 2.4 megapascals. Remarkably, the PLA/PBAT mixes containing 5% silica nanoparticles (M3) exhibited the most exceptional qualities.

Nanoindentation is a widely used method for evaluating the mechanical characteristics of materials. The Oliver–Pharr method is the most frequently used technique for determining the hardness and elastic modulus of a material by nanoindentation. The approach involves determining the nanoindentation hardness (H) by relating it to the ultimate penetration depth of the indent. This may be performed using the following formula

*H* = *P*max/*A*

where *P*max is the maximum applied load measured at the maximum depth of penetration (*h*max), and *A* is the contact area.

**Figure 10:**
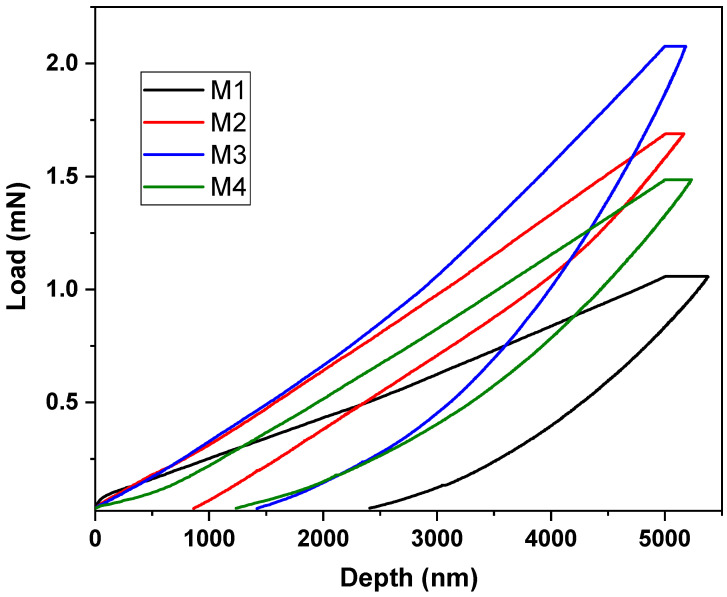
Indentation load vs. depth curves of PBAT/PLA-based multifunctional electrospun fabrics.

**Figure 11:**
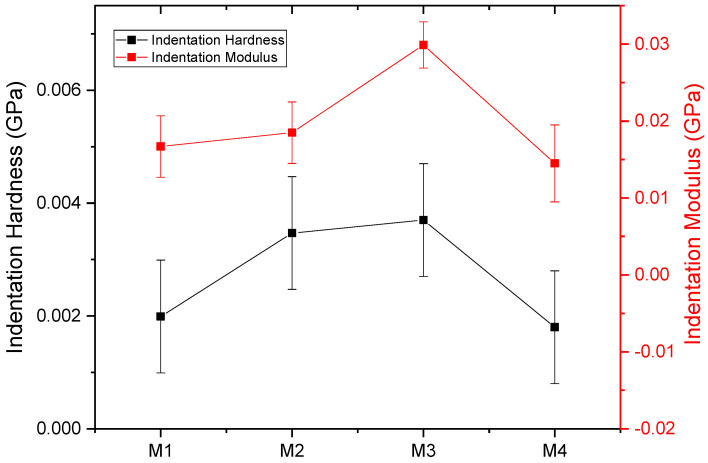
Indentation hardness vs. modulus of PBAT/PLA-based multifunctional electrospun fabrics.

In order to examine the mechanical characteristics more thoroughly, the nanoindentation method was used. Figure 10 displays the curves depicting the relationship between the load applied to the PBAT/PLA-based multifunctional electrospun textiles and the corresponding depth of indentation. The samples’ indentation hardness versus the modulus measurements exhibited comparable tendencies. The specific values obtained for the hardness and modulus from the indentation load versus depth curves are provided in Table 2 and shown in Figure 11.

## 4. Conclusions

This article describes the creation of a protective cloth particularly for handicapped people. The extremely hydrophobic fabric was created using PBAT/PLA biobased polymers with an average diameter of roughly 700–950 nm and incorporated silica nanoparticles. The fabric’s porosity promotes breathability, while the silica nanoparticles work as efficient infrared reflectors, keeping the user cool on hot days. This protective apparel resulted in a maximum 24% decrease in the floor’s temperature under direct sunshine. Superhydrophobic textiles (contact angle of ~154°) have the ability to repel water and keep the users dry. The fabric had excellent mechanical qualities, including a maximum ultimate tensile strength of 2.4 MPa. The highly hydrophobic fabric incorporating SiO_2_ nanoparticles effectively blocked UV radiation in both zones; the area-blocking percentages for UV-A (315–400 nm) and UV-B (280–315 nm) were 74% and 78%, respectively. The developed protective fabric outperformed in terms of reflection (%) because reflection of UV rays in the 280–400 range not only keeps the body cool but also helps to avoid skin burns and aging, both of which contribute to skin cancer. The fabric is appropriate for a variety of goods, including adult diapers, outdoor clothing, and accessories for people with impairments. The article also underlines the need for choosing suitable materials for garment protectors, notably bibs or feeding aprons, as well as the mobility, eating, and dressing issues that handicapped people encounter.

## Figures and Tables

**Table 1:** PBAT/PLA solution containing silica nanoparticles.

Nanofibrous Electrospun Fabric Samples’ Codes	PBAT Concentration (% *w*/*v*)	PLA Concentration (% *w*/*v*)	SiO_2_ Concentration (% *w*/*w*)
M1	15	12	1
M2			3
M3			5
M4			7

**Table 2:** Nanoindentation hardness and modulus values obtained from the indentation load vs. depth curves.

Sample	Indentation Hardness (GPa)	Indentation Modulus (GPa)
M1	0.00199	0.0167
M2	0.00347	0.0185
M3	0.00370	0.0299
M4	0.00180	0.0145

## Data Availability

Data are contained within the article.
